# Effects of Irrigation Water Amount and Humic Acid on β-Glucan Synthesis in Post-Anthesis Grains of Naked Oats

**DOI:** 10.3390/life15030343

**Published:** 2025-02-21

**Authors:** Chunxiang Sun, Qi Wang, Wen Sun, Junying Wu, Shihua Gao, Yandi Liu, Baoping Zhao

**Affiliations:** 1 College of Agriculture, Inner Mongolia Agricultural University, Hohhot 010018, China; 2 Agricultural and Animal Husbandry Sciences Academy of Tongliao, Tongliao 028000, China; 3 Agriculture and Animal Husbandry Science Technology Bureau of Zhuozi County, Ulanqab 012000, China; 4 College of Vocational and Technical, Inner Mongolia Agricultural University, Baotou 014109, China

**Keywords:** oat, irrigation amount, humic acid, β-glucan, post-anthesis

## Abstract

Naked oats offer substantial nutritional and health benefits, primarily due to their main dietary fiber component, soluble β-(1,3)(1,4)-D-glucan (β-glucan). In a pool experiment, humic acid (HA) was applied once during both the booting and anthesis stages at varying irrigation amounts (60 mm, 120 mm, and 180 mm) to assess changes in β-glucan content in grains post-anthesis. Results indicated that at 5 days post-anthesis (DPA), the β-glucan content (3.14% W/W) in grains increased by 16%with the application of HA, compared to the control treatment of spraying an equal volume of water (*p* < 0.01). The β-glucan content (4.13%, 4.51%) at 15 and 25 DPA reflects increases of 9% and 5% compared to the control. Overall, the application of HA enhanced the β-glucan content in grains, with levels gradually increasing at 5, 15, and 25 DPA; however, the amplitude of the increase gradually declined over time. The β-glucan content in grains at 5 and 15 DPA, along with glucose content in panicles at 20 DPA, directly influenced the β-glucan content in grains at 25 DPA. At 10 DPA, the distribution of sucrose in the leaves and panicles influences the soluble sugar content, subsequently regulating the β-glucan content in the grains at 15 DPA. Specifically, the sucrose content in the leaves exerts a positive regulatory effect, whereas in the panicles exerts a negative regulatory effect.

## 1. Introduction

Oat (*Avena sativa* L.) is a member of Poaceae and is an important dual-purpose crop for both food and fodder. Oats are classified into two main types based on the presence of hulls: hulled oats and naked oats (*Avena nuda* L.). In Europe and North America, oat cultivation primarily focuses on hulled oats, which are the subject of most research. In contrast, China is the center of origin for large-grained naked oats, which have a long history of cultivation. These oats are predominantly grown in major producing regions of oats [1]. Oats are nutrient-dense and are regarded as one of the best sources of complete nutrition among grains. Numerous domestic and international studies indicate that oats can lower blood lipids and cholesterol without adverse effects. Additionally, oats help regulate immune function, improve resistance, and mitigate diabetes [2,3]. Current research suggests that the health benefits of oats primarily arise from their dietary fiber component, soluble β-glucan [4]. β-glucanis a polysaccharide located in the cell walls of cereal grains, particularly in barley and oats [5]. As the primary functional component of oats, β-glucan-rich products offer substantial health benefits [6]. Naked oats are generally believed to contain higher levels of β-glucan than hulled oats [7,8]. Therefore, increasing the β-glucan content in naked oats is a key focus of cultivation research because of their significant nutritional and health benefits.

Humic substances are materials derived from the decomposition of plant, animal, and microbial residues and from the metabolic activity of soil microorganisms that have been extensively transformed since their production [9]. Operationally, they can be separated and classified into the following fractions: fulvic acids (soluble in acid and alkaline pH), humic acids (insoluble at acid pH and soluble at alkaline pH), and humin (insoluble at acid and alkaline pH) [10]. HA is a natural organic polymer compound and a major component of humus [11]. The application of HA on plants such as maize, cabbage, and flue-cured tobacco showed that HA enhanced growth and development, improved stress resistance, increased quality, and boosted yield for certain crops [12,13,14,15]. Previous research shows that water-soluble HA foliar fertilizers significantly enhance the biological traits of wheat, promote plant growth, improve commercial characteristics [16], and increase carbohydrate content [17]. Additionally, the application of these fertilizers to rice enhances growth attributes, yield, and mineral content [18,19]. Foliar spraying with an appropriate amount of water-soluble HA fertilizer enhances the biological traits of oats, increases the number of panicles, grains per panicle, crude protein on a dry matter basis, and grain and forage yields [20].

Drought is one of the most serious stresses affecting crops and may also have a considerable effect on the chemical composition of the grain, including the storage protein (gliadins, glutenins) and dietary fiber (arabinoxylan, β-glucan) content and composition [21,22]. Generally, drought stress is known to reduce the carbohydrate content (including sucrose and starch) of the grain [23,24] and to increase the protein content. However, the effects are highly dependent on the degree and timing of the drought and on interactions with other environmental stresses [25]. Research shows that drought stimulates the synthesis of β-glucan in oats [26]. However, a deficiency in soil moisture post-anthesis can result in a reduction in β-glucan content [27]. Whereas other studies suggest that irrigation does not affect β-glucan synthesis [28]. This implies that the effect of moisture on β-glucan content in oats remains unclear. Improving the stability of grain quality and quantity under drought conditions is important cultivation research.

Currently, the literature on the application of HA to oats is scarce, with a primary focus on its effects on fodder and grain yield. Limited research exists on the impact of HA on β-glucan, a key quality indicator of oat grains. This study aims to investigate the impact of HA application during the booting and anthesis stages on oat grains under varying irrigation amounts. A pool experiment will be conducted to assess the changes in β-glucan content in oat grains following HA treatment. The effects of soluble sugar, sucrose, glucose, and fructose content on β-glucan content in oat grains will be analyzed across treatments, to elucidate the mechanisms by which HA promotes β-glucan synthesis. The findings of this study will provide theoretical insights and practical guidance for enhancing oat quality.

## 2. Materials and Methods

### 2.1. Study Site

A pool experiment was carried out from April to August 2017. Each pool was 3.5 m-long, 4 m-wide and 1.5 m-high (overhead rainproof shed). The experimental soil was collected from an oat field (40°30′ N and 110°33′ E) in Salaqi, Inner Mongolia, China, at the Vocational and Technical College of Inner Mongolia Agricultural University’s Science and Technology Park. The soil type was classified as Inceptisol (USDA Soil Taxonomy). Sandy loam was the type of soil used in the experiment [29]. The 0–20 cm soil layer prior to the start of the experiment had the following characteristics: soil pH 8.02, organic matter 15.65 g kg^−1^, and available N, P, K were35.10 mg kg^−1^, 19.50 mg kg^−1^, and 75.14 mg kg^−1^, respectively. Meteorological data of the study site in 2017 were as follows: average temperature of 7.1 °C and sunshine duration of 3056.3 h.

### 2.2. Experimental Design and Set-Up

The research used the split-plot design, with the main plots consisting of three irrigation amounts: 60 mm, 120 mm, and 180 mm, which were controlled using a water meter, and irrigated at jointing, heading, and filling stages (1:2:1). The sub-plots contained two foliar applications: HA (diluted 500-fold) and water, with a spray amount of 1.5 L ha^−1^ at the booting and heading stages. The HA liquid fertilizer from Inner Mongolia Yongye Biotechnology Co., Ltd. (Hohhot, China), contained the following: HA 50 g L^−1^, N + P + K ≥ 200 gL^−1^, and trace elements (Mn, Mo, Zn, etc.) ≥ 10 g L^−1^. Every treatment was conducted in three replicates. In this study, materials were selected on the naked oat cultivar Mengnong Dayan No.1, which was planted through manual strip sowing (April 2017) with a row spacing of 20 cm. The experimental plot area was 14 m^2^, with a seeding density of 150 kg ha^−1^. Field management was conducted according to local oat cultivation and management practices.

### 2.3. Sampling and Handling

After the flowering oat plants, selected segments with uniform growth within the plot were collected at 5, 10, 15, 20, and 25 DPA. The harvested plants were placed on ice, and the stem, leaf, panicle, and grain were separated within one hour. Thereafter, we analyzed the glucose and fructose content of the oats that had been frozen in liquid nitrogen and stored at −80 °C. The remaining samples were blanched at 105 °C for 30 min, then dried at 80 °C to a constant weight to determine soluble sugars, sucrose, and β-glucan content.

### 2.4. Analytical Procedures

#### 2.4.1. Quantification of Dry Matter

After the flowering oat plants, selected segments with uniform growth within the plot were collected at 5, 15, and 25 DPA. The stem, leaf, and panicle were separated in the laboratory, and the samples were blanched at 105 °C for 30 min, then dried at 80 °C to a constant weight to calculate the dry weight.

#### 2.4.2. Quantification of β-Glucan Content

The sampled oat grains at 5, 15, and 25 DPA were dried at 80 °C in an oven, thoroughly ground into flour with a grinder, and then stored in a dryer before β-glucan content measurement. β-glucan content was determined using the mixed-linkage β-glucan assay kit (Yiguo, Shanghai, China), and the experiment was performed according to the kit instructions.

#### 2.4.3. Quantification of Soluble Sugars and Sucrose Content

The sampled oat stem, leaf, and panicle at 5, 10, 15, 20, and 25 DPA were dried at 80 °C in an oven, thoroughly ground into flour with a grinder, and then stored in a dryer before soluble sugars content measurement. The soluble sugar content was determined using the anthrone method [30]. The sucrose content was determined using the resorcinol method [31].

#### 2.4.4. Quantification of Glucose and Fructose Content

The sampled oat stem, leaf, and panicle at 5, 10, 15, 20, and 25 DPA were frozen in an ultra-low temperature freezer and held at −80 °C. They were then thoroughly ground into flour with liquid nitrogen before measuring glucose and fructose content. The glucose and fructose contents were determined using the high-performance liquid chromatography (HPLC) method [32].

### 2.5. Statistical Analysis

The data were organized using Microsoft Excel (Version 2019), and a two-way analysis of variance (ANOVA) was carried out using IBM SPSS Statistics (Version 22.0). Significant differences were then compared using Fisher’s Least Significant Difference (LSD) test at a significant level of *p* = 0.05. Graphs were generated using Origin (Version 2021). A random forest model was developed using the random Forest package in R Studio (Version 4.4.1), and structural equation modeling (SEM) analysis was performed with the Lavaan package in R Studio (Version 4.4.1).

## 3. Results

### 3.1. Dry Matter Profiling

**Figure 1:**
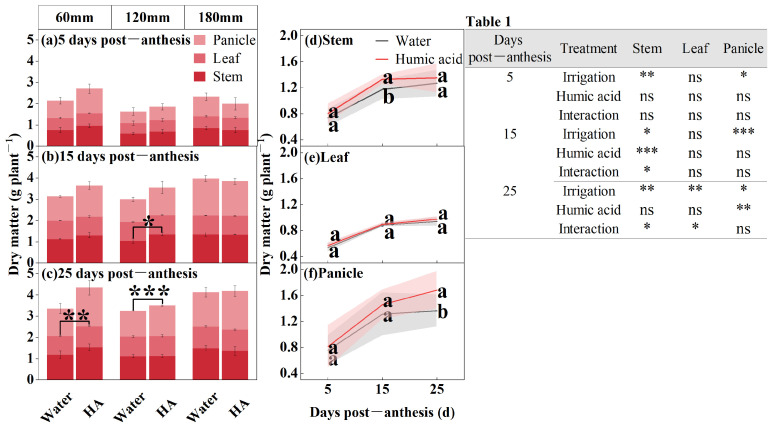
Dry matter of oat stem, leaf, and panicle under two foliar applications (HA and water) with three irrigation amounts (60 mm, 120 mm, and 180 mm) at 5, 15, and 25 DPA (**a**–**c**). Dry matter of oat stem, leaf, and panicle under two foliar applications (HA and water) at 5, 15, and 25 DPA (**d**–**f**). Combined analysis of variance for parameters of irrigation amount and foliar application, dry matter of oat stem, leaf, and panicle at 5, 15, and 25 DPA (Table 1). Values are means ± standards deviation (*n* = 3, (**a**–**c**)) (*n* = 9, (**d**–**f**)). Different lowercase letters with the column under the same DPF mean significant differences at *p* < 0.05 by Fisher’s Least Significant Difference (LSD) test. * *p* < 0.05, ** *p* < 0.01, *** *p* < 0.001, and ns no significant difference.

In order to study the accumulation pattern of dry matter in the aboveground organs of oats during the filling period (5, 15, and 25 DPA), the researchers determined the dry matter in foliar applications (HA and water) at different irrigation amounts (60 mm, 120 mm, and 180 mm) (Figure 1). The amount of irrigation significantly influences the dry matter in the stems and panicles of oats from 5 to 25 DPA, and the leaves at 15 DPA. HA significantly influences the stems’ dry matter at 15 DPA and the leaves at 25 DPA. The interaction between the irrigation amount and HA significantly influences the stems’ dry matter at 15 and 25 DPA, as well as in the leaves at 25 DPA. The dry matter over time post-anthesis was gradually increasing (Figure 1a–c). In 60 mm and 120 mm irrigation amounts, the application of HA enhances the dry matter in oats. Under 60 mm irrigation, the application of HA resulted in 12%increase in leaf dry matter at 25 DPA compared to the control (*p* < 0.01). Under 120 mm irrigation, stem dry matter increased by 29% (*p* < 0.05) at 15 DPA and 19% (*p* < 0.001) in panicle at 25 DPA. From 5 to 25 DPA, the application of HA enhances the dry matter in the aboveground organs of oats (Figure 1d–f). The application of HA raised the stem dry matter to 1.33 g plant^−1^ at 15 DPA, representing a 13% increase compared to the spraying water (*p* < 0.05) (Figure 1d). The application of HA enhances the panicle dry matter in oat. However, this enhancement generally rises as the number of days post-anthesis increases (Figure 1f). The application of HA raised the panicle dry matter to 1.68 g plant^−1^ at 25 DPA, representing a 23% increase compared to the spraying water (*p* < 0.05) (Figure 1f).

### 3.2. β-Glucan Content Profiling

**Figure 2:**
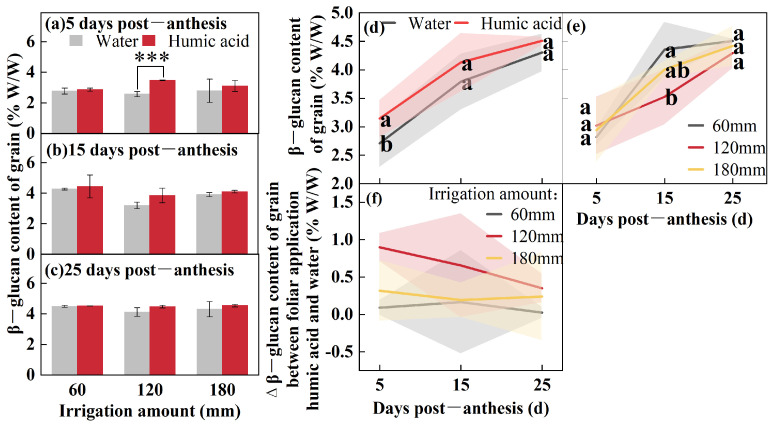
β-glucan content of oat grains under two foliar applications (HA and water) with three irrigation amounts (60 mm, 120 mm, and 180 mm) at 5, 15, and 25 DPA (**a**–**c**). β-glucan content of oat grains under two foliar applications (HA and water) at 5, 15, and 25 DPA (**d**). β-glucan content of oat grains under three irrigation amounts (60 mm, 120 mm, and 180 mm) at 5, 15, and 25 DPA (**e**). Δβ-glucan content from foliar application of humic acid and water under three irrigation amounts (60 mm, 120 mm, and 180 mm) of oat grains at 5, 15, and 25 DPA (**f**). Values are means ± standards deviation (*n* = 3, (**a**–**c**,**f**)) (*n* = 9, (**d**,**e**)). *** *p* < 0.001. Different lowercase letters with the column under the same DPF mean significant differences at *p* < 0.05 by Fisher’s Least Significant Difference (LSD) test.

To study the accumulation pattern of β-glucan in oat grains during the filling period (5, 15, and 25 DPA), the β-glucan content from the foliar application (HA and water) was determined under different irrigation amounts (60 mm, 120 mm, and 180 mm) (Figure 2). The β-glucan content at 5 DPA was influenced by HA (*p* < 0.05), while at 15 DPA it was influenced by the irrigation amount (*p* < 0.01) (Table 1). The β-glucan content increased gradually over time post-anthesis (Figure 2a–c). Under 60 mm irrigation, the application of HA resulted in a 3%, 4%, and 1% increase in β-glucan content at 5, 15, and 25 DPA compared to the spraying of water. Under 120 mm irrigation, the β-glucan content increased by 35% (*p* < 0.001), 8%, and 5%at 5, 15, and 25 DPA, respectively, compared to thecontrol. Under 180 mm irrigation, the β-glucan content increased by 21%, 11%, and 6%. The three irrigation treatments were combined to analyze the differences in β-glucan content among different foliar spray treatments (Figure 2d). The results indicated that using HA resulted in a β-glucan content of 3.14% W/W at 5 DPA, representing a 16% increase compared to the spraying of water (*p* < 0.01). The β-glucan content from foliar application of HA at 15 and 25 DPA was 4.13% W/W and 4.51% W/W, respectively, which were 9% and 5% higher than the control. The two foliar spray treatments were combined to compare the differences in β-glucan content among different irrigation amounts (Figure 2e). At 15 DPA, the β-glucan content under 60 mm irrigation was 4.36% W/W, which was 24% higher than that under 120 mm irrigation (*p* < 0.05). Across all irrigation amounts, the application of HA enhanced the β-glucan content in grains. However, this enhancement generally declined as the number of days post-anthesis increased (Figure 2f).

**Table 1:** Combined analysis of variance for parameters of irrigation amount and foliar application include soluble sugar, sucrose, glucose, fructose, and β-glucan content of oat stem, leaf, and panicle at 5, 10, 15, 20, and 25 DPA.

Days Post-Anthesis	Treatment	Soluble Sugar Content			Sucrose Content			Glucose Content			Fructose Content			β-Glucan Content
		Stem	Leaf	Panicle	Stem	Leaf	Panicle	Stem	Leaf	Panicle	Stem	Leaf	Panicle	Grain
**5**	Irrigation amount	ns	*	***	***	***	***	***	***	***	***	***	***	ns
	Humic acid	ns	**	**	ns	***	***	***	***	*	ns	***	ns	*
	Interaction	ns	ns	ns	ns	*	ns	***	*	ns	ns	**	ns	ns
**10**	Irrigation amount	***	***	***	***	***	***	***	***	ns	**	***	ns	-
	Humic acid	**	**	**	***	***	ns	***	***	***	***	**	ns	-
	Interaction	ns	**	ns	ns	ns	ns	*	*	ns	ns	ns	ns	-
**1** **5**	Irrigation amount	***	**	***	**	***	***	***	***	***	**	ns	***	**
	Humic acid	*	*	***	ns	ns	***	**	ns	**	ns	ns	ns	ns
	Interaction	ns	ns	ns	ns	ns	***	*	ns	*	ns	ns	ns	ns
**20**	Irrigation amount	*	***	*	**	***	ns	ns	ns	ns	ns	ns	ns	-
	Humic acid	**	**	**	**	ns	***	*	**	ns	ns	*	ns	-
	Interaction	*	*	**	ns	ns	ns	ns	ns	ns	ns	ns	ns	-
**2** **5**	Irrigation amount	***	***	*	***	***	***	***	***	**	***	***	**	ns
	Humic acid	***	***	**	*	***	***	***	***	ns	***	***	ns	ns
	Interaction	ns	***	ns	ns	***	ns	***	***	ns	***	*	ns	ns

* *p* < 0.05, ** *p* < 0.01, *** *p* < 0.001, and ns no significant difference.

### 3.3. Soluble Sugar Content Profiling

**Figure 3:**
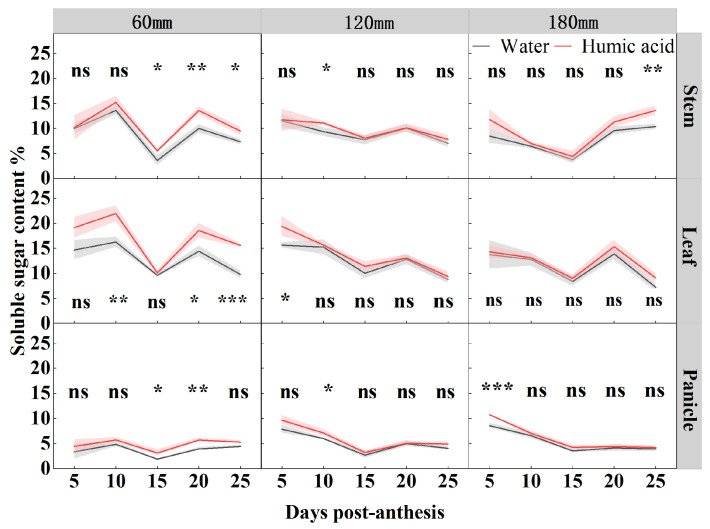
Soluble sugar contents of oat stem, leaf, and panicle under two foliar applications (HA and water) with three irrigation amounts (60 mm, 120 mm, and 180 mm) at 5, 10, 15, 20, and 25 DPA. Values are means ± standard deviation (*n* = 3). * *p* < 0.05, ** *p* < 0.01, *** *p* < 0.001, and ns no significant difference.

In order to study the accumulation and distribution pattern of soluble sugar in oat stem, leaf, and panicle during the filling period (5, 10, 15, 20, and 25 DPA), researchers determined the soluble sugar content in foliar applications (HA and water) at different irrigation amounts (60 mm, 120 mm, and 180 mm) (Figure 3). Both the irrigation levels and HA significantly influenced the soluble sugar content of oat leaves and panicles from 5 to 25 DPA, and of stems from 10 to 25 DPA. The interaction between irrigation levels and HA significantly influenced the soluble sugar content of oat leaves at 10 and 25 DPA, as well as that of stems, leaves, and panicles at 20 DPA (Table 1). Under 60 mm irrigation, the soluble sugar content of oats peaked at 10 and 20 DPA during the period of 5–25DPA. In contrast, under 120 mm and 180 mm irrigation, the soluble sugar content of oats gradually declined from 5 to 15 DPA and peaked at 20 DPA during the period of 5–25DPA. The application of HA under 60 mm irrigation significantly enhanced the soluble sugar content of oat stems at 15, 20, and 25 DPA by 54%, 36%, and 29%, respectively, compared to water. The soluble sugar content of the leaves increased by 35%, 29%, and 60% at 10, 20, and 25 DPA, respectively. Additionally, the soluble sugar content of panicles increased by 66% and 46% at 15 and 20 DPA, respectively. Under 120 mm irrigation, the application of HA enhanced the soluble sugar content of oat leaves at 5 DPA by 24% (*p* < 0.05). Furthermore, the soluble sugar content of stems and panicles at 10 DPA increased by 19% (*p* < 0.05). Under 180 mm irrigation, the soluble sugar content of panicles increased by 25% (*p* < 0.001) at 5 DPA, while the soluble sugar content of stems increased by 31% (*p* < 0.01) at 25 DPA.

### 3.4. Content of Sucrose, Glucose, and Fructose Profiling

**Figure 4:**
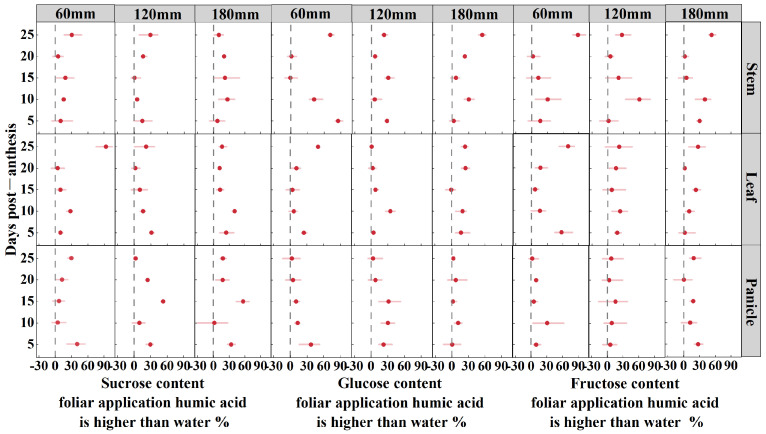
Sucrose, glucose, and fructose content in foliar application humic acid is higher than water under three irrigation amounts (60 mm, 120 mm, and 180 mm) of oat stem, leaf, and panicle at 5, 10, 15, 20, and 25 DPA.

The sucrose, glucose, and fructose content from foliar application of HA was found to be higher than that of water under three irrigation amounts (60 mm, 120 mm, and 180 mm) for oat stem, leaf, and panicle at 5, 10, 15, 20, and 25 DPA (Figure 4).

The amount of irrigation significantly influences the sugar content in the stems, leaves, and panicles of oats from 5 to 25 DPA, with the exception of the panicles at 20 DPA. HA significantly influences the sugar content in the stems at 10, 20, and 25 DPA, in the leaves at 5, 10, and 25 DPA, and in the panicles at 5, 15, 20, and 25 DPA. The interaction between irrigation amount and HA significantly influences the sugar content in the leaves from 5 to 25 DPA, as well as in the panicles at 15 DPA (Table 1). Under 60 mm of irrigation, HA increased the sugar content in the leaves of oats by 29% compared to water. Conversely, under 120 mm and 180 mm of irrigation, HA increased the sugar content in the panicles by 24% and 26%, respectively. At 5 DPA, the application of HA with 60 mm and 180 mm of irrigation significantly increased the sugar content in the panicles by 41% and 34%, respectively, whereas 120 mm of irrigation primarily increased the sugar content in the leaves by 31%. At 10 DPA, all irrigation levels primarily increased the sugar content in the leaves by 16% to 41%. At 15 DPA, 120 mm and 180 mm of irrigation significantly increased the sugar content in the panicles by 53% and 57%, respectively, whereas 60 mm of irrigation primarily increased the sugar content in the stems by 19%. At 20 DPA, 60 mm and 120 mm of irrigation significantly increased the sugar content in the panicles by 13% and 25%, respectively, whereas 180 mm of irrigation primarily increased the sugar content in the stems by 20%. Overall, this indicates that the application of HA under higher irrigation levels tends to favor sugar accumulation in the panicles and stems at later stages. At 25 DPA, 60 mm, 120 mm, and 180 mm of irrigation primarily increased the sugar content in the leaves, stems, and panicles by 93%, 29%, and 18%, respectively. This suggests that the application of HA leads to a notable increase in sugar accumulation in leaves under lower irrigation conditions.

The glucose content in the stems, leaves, and panicles of oats at 5, 15, and 25 DPA, as well as in the stems and leaves at 10 DPA, was influenced by irrigation amount (*p* < 0.01). The glucose content in the stems and leaves from 5 to 25 DPA, except for the leaves at 15 DPA, and in the panicles from 5 to 15 DPA, was significantly affected by HA. The glucose content in the stems and leaves from 5 to 25 DPA, except for the stems at 20 DPA and the leaves at 15 and 20 DPA, was significantly affected by interaction; additionally, the panicles at 15 DPA were influenced by interaction (*p* < 0.05) (Table 1). Under irrigation amounts of 60 mm and 180 mm, HA primarily increased glucose content in the stems post-anthesis by 40% and 23%, respectively, compared to that of water. At 120 mm, HA primarily increased glucose content in the panicles by 19%. The application of HA at 5 DPA, with irrigation amounts of 60 mm and 120 mm, primarily increased glucose content in the stems by 85% and 28%, respectively, while 180 mm increased glucose content in the leaves by 17%. At 10 DPA, irrigation amounts of 60 mm and 180 mm increased glucose content in the stems by 42% and 30%, respectively, while 120 mm increased glucose content in the leaves by 35%. At 15 DPA, irrigation amounts of 60 mm and 120 mm increased glucose content in the panicles by 10% and 32%, respectively, while 180 mm increased glucose content in the stems by 7%. At 20 DPA, irrigation amounts of 60 mm and 180 mm increased glucose content in the leaves by 11% and 25%, respectively, while 120 mm increased glucose content in the panicles by 8%. Finally, at 25 DPA, all irrigation levels increased glucose content in the stems by 23% to 71%. This demonstrates that the application of HA affects the increase of stem glucose content even at the late growth stage.

The fructose content in the stems, leaves, and panicles at 5 and 25 DPA, in stems and leaves at 10 DPA, as well as in stems and panicles at 15 DPA, was influenced by the irrigation amount (*p* < 0.01). The fructose content in leaves at 5 and 20 DPA, as well as in stems and leaves at 10 and 25 DPA, was significantly affected by HA. The fructose content in leaves at 5 and 25 DPA, as well as in stems at 25 DPA, was significantly affected by the interaction (Table 1). The irrigation amount of 60 mm, combined with HA, increased the fructose content in oat leaves by 34% compared to using water. At irrigation amounts of 120 mm and 180 mm, HA enhanced the fructose content in stems by 23% and 26%, respectively. Following the application of HA, at 5 DPA, irrigation amounts of 60 mm and 120 mm enhanced the fructose content in leaves by 57% and 18%, respectively, while 180 mm primarily enhanced the fructose content in stems by 30%. At 10 DPA, the application of HA primarily enhanced the fructose content in stems by 31% to 60% at all irrigation levels. At 15 DPA, irrigation amounts of 60 mm and 120 mm enhanced the fructose content in stems by 13% and 21%, respectively, while 180 mm primarily enhanced the fructose content in leaves by 23%. At 20 DPA, the application of HA primarily enhanced the fructose content in leaves by 2% to 17% at all irrigation levels. At 25 DPA, the application of HA primarily enhanced the fructose content in stems by 27% to 89% at all irrigation levels.

### 3.5. Relative Importance of Variables of RF Models on the β-Glucan Content

**Figure 5:**
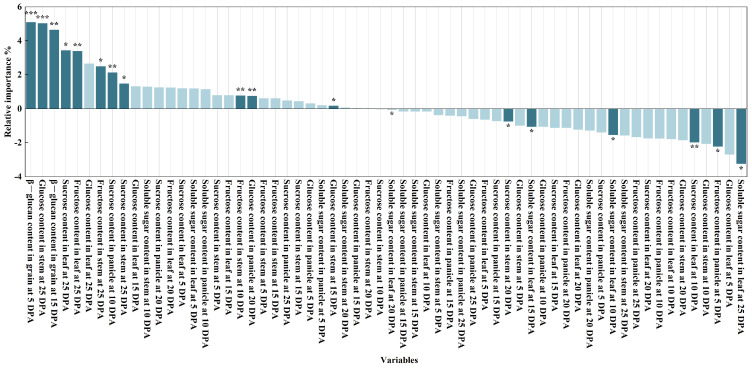
Relative importance of variables of RF models on the β-glucan content in grain at 25 DPA. * *p* < 0.05, ** *p* < 0.01, *** *p* < 0.001.

This study examines the effects of HA application on sugar content in various organs post-anthesis and its impact on the β-glucan content of mature grains. The study identified key factors influencing the levels of soluble sugars, sucrose, glucose, and fructose in various organs under varying irrigation levels in the days post-anthesis. Using the random forest model, the researchers discovered 18 indicators of significant influencing factors (Figure 5). The identified factors include: β-glucan content in grains at 5 DPA, glucose content in stems at 25 DPA, β-glucan content in grains at 15 DPA, sucrose content in leaves at 25 DPA, fructose content in leaves at 25 DPA, fructose content in stems at 25 DPA, sucrose content in panicles at 10 DPA, sucrose content in stems at 25 DPA, fructose content in stems at 10 DPA, glucose content in panicles at 20 DPA, glucose content in stems at 15 DPA, soluble sugar content in leaves at 20 DPA, sucrose content in stems at 20 DPA, soluble sugar content in leaves at 15 DPA, soluble sugar content in leaves at 10 DPA, sucrose content in leaves at 10 DPA, fructose content in panicles at 5 DPA, and soluble sugar content in leaves at 25 DPA. These traits are the primary determinants of β-glucan content at grain maturity.

### 3.6. Structural Equation Model (SEM) for β-Glucan Content

**Figure 6:**
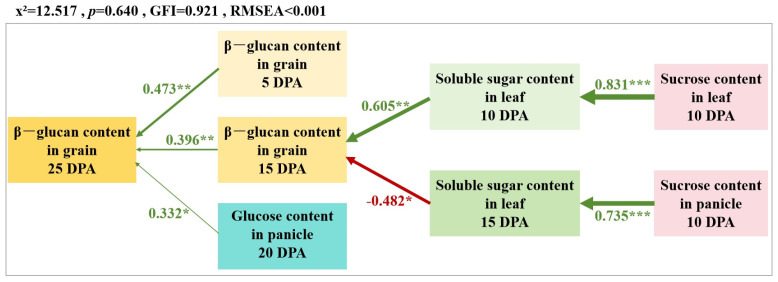
Construction of structural equation model (SEM) for β-glucan content in grain at 25DPA under irrigation amount and HA. Square boxes represent the variables applied in the model. Green and red real line arrows showed significant positive and negative correlation, respectively. Thickness of the arrow indicated the strength of the influence relationship. Numbers adjacent to arrows were the standardized path coefficients. * *p* < 0.05, ** *p* < 0.01, and *** *p* < 0.001.

To elucidate the direct and indirect effects of various indicators, the researchers constructed a structural equation model. This model connects several factors: β-glucan in grains at 5 and 15 DPA, glucose in panicles at 20 DPA, soluble sugars in leaves at 10 and 15 DPA, and sucrose in leaves and panicles at 10 DPA to β-glucan at the grains’ maturity stage, demonstrating a good fit (*p* = 0.640). The aim of this study was to investigate the interactions among these indicators through various pathways and their effects on β-glucan at the grain maturity stage (Figure 6). The results indicated that β-glucan in grains at 5 and 15 DPA, along with glucose in panicles at 20 DPA, directly influences β-glucan levels at the grain maturity stage. These factors exhibit significant positive effects, with path coefficients of 0.473, 0.396, and 0.332, respectively. The distribution of sucrose in leaves at 10 DPA affects soluble sugar content, positively regulating β-glucan levels in grains at 15 DPA. In contrast, the distribution of sucrose in panicles at 10 DPA negatively regulates β-glucan levels in grains by influencing soluble sugar content in leaves at 15 DPA.

## 4. Discussion

### 4.1. β-Glucan Content Response to Irrigation Amount

Increasing the β-glucan content in naked oats is a primary focus of cultivation research due to its substantial nutritional and health benefits. Qi et al. [33] demonstrated that the β-glucan content in oats is influenced by multiple genetic and environmental factors. Variations in β-glucan content across diverse environments primarily result from differences in grain-filling conditions [34]. Fastnaught et al. [8] proposed that low humidity and cool conditions during grain maturation promote β-glucan accumulation. Numerous studies indicate that quality indicators, including soluble sugars, soluble solids, titratable acidity, and vitamin C content, are significantly influenced by moisture levels, with increased moisture resulting in a decline in these indicators [35]. In this study, irrigation amounts influenced the β-glucan content in oat grains at 15 DPA. The β-glucan content under 60 mm irrigation was 4.36% W/W, 24% higher than that under 120 mm irrigation (*p* < 0.05), with higher irrigation correlating with reduced β-glucan content. This is consistent with the results of We et al. [36] in highland barley, which indicate that rainfall has an inhibitory effect on β-glucan. However, the study suggests that there is a certain threshold; under the context of arid conditions in the Tibetan Plateau, increasing annual rainfall appropriately, within 600 mm, is beneficial for the rise in β-glucan content. Increased moisture dilutes nutrients in the plant’s harvesting organs, while osmotic regulation under varying moisture conditions may also modify nutrient concentrations [37,38]. Under moisture stress, heightened activity of cell wall-modifying enzymes may facilitate the influx of hexoses into storage cells or accelerate sucrose transport from the phloem to the apoplast via sucrose concentration gradients, generating glucose signals that promote cell growth and sugar accumulation [35]. This may also elucidate why, under low irrigation conditions, the β-glucan content in grains was comparatively high in this study. The research by Marianna Rakszegi et al. [39] on wheat indicates that drought stress reduces the β-glucan content in seeds, which contradicts the results of this experiment. It is evident that the effect of moisture on grain β-glucan is uncertain, and this experiment lacks exploration of the irrigation threshold.

### 4.2. β-Glucan Content Response to Foliar Application Humic Acid

Previous studies have indicated that foliar application of humic acid in flax crops enhances parameters such as polysaccharide content, total phenols, and biological, seed, oil, and percentage yield [40]. Additionally, in olive trees, increases in chlorophyll, carbohydrates, proteins, fiber, and fat content were observed [41], demonstrating the beneficial effects of HA on plant quality. Zhang et al. [34] found that β-glucan content primarily accumulates in the week before maturity, while adverse environmental conditions in later stages may reduce this content. This study’s results indicate that spraying HA can increase β-glucan content in oat grains and the dry matter in aboveground organs, with the highest levels observed at maturity. Notably, a significant increase in β-glucan content was observed at 5 DPA; however, the amplitude of increase at 15 and 25 DPA gradually declined. Whereas, from 5 to 25 DPA, the dry matter increased in the panicle, and the amplitude gradually rose; at 25 DPA, the dry matter increased significantly. Therefore, it can be seen that the application of HA has an enhancing effect on both the biomass of the panicle and the β-glucan content in naked oats. This is consistent with the findings of Shen et al., Adnan et al., and Doroodian et al., which indicate that applying HA to foxtail millet, maize, and wheat can enhance crop yields [11,42,43]. However, the accumulation processes of the two are mutually inhibitive. In the early grain-filling stage, specifically, 5–15DAF, HA mainly increases the β-glucan content in the grains, while in the later grain-filling stage, which is 15–25DAF, the effect of HA is primarily reflected in the growth of panicle biomass, with a reduced increase in β-glucan content.

### 4.3. Effects of Carbohydrates on β-Glucan Formation in Oat Grains

β-glucans are unsubstituted, unbranched polysaccharides of β-D-glucopyranosyl monomers polymerized through (1,3)- and (1,4)-linkages. As a polysaccharide, β-glucan should be closely associated with assimilation, in particular, glucose metabolism during grain development. The inverse relationship between starch and β-glucan content has been documented [44]. Geng et al. [45] indicated that genes involved in carbohydrate metabolism, as well as starch and sucrose metabolism, play important roles in β-glucan synthesis. Photosynthesis provides the energy and carbon sources for the synthesis of β-glucan and starch, thereby positively regulating the accumulation of β-glucan in grains. While starch and sucrose may compete with β-glucan for glucose, they can also be metabolized into glucose donors for β-glucan synthesis. In this study, a proposed model for β-glucan synthesis in oat grain was developed. The β-glucan content of grains at5 and 15 DPA, along with glucose in panicles at 20 DPA, directly influences β-glucan levels at the grain maturity stage. At 10 DPA, the distribution of sucrose in the leaves and panicles influences the soluble sugar content, subsequently regulating the β-glucan content in the grains at 15 DPA. Specifically, the sucrose content in the leaves exerts a positive regulatory effect, whereas the sucrose content in the panicles exerts a negative regulatory effect. This phenomenon may be attributed to HA’s ability to enhance the photosynthetic characteristics of plants [46]. Leaves assimilate inorganic substances into organic matter, which is then transported as sucrose within the plant. At 15 DPA, when sucrose is distributed to the panicles, it decomposes into glucose and fructose, which are utilized for starch synthesis. Competition for substrates between glucose used for starch synthesis and β-glucan synthesis may limit the increase in β-glucan content. At 20 DPA, most glucose in the panicles is used for starch synthesis, further limiting the increase in β-glucan content in the grains at maturity. In summary, the foliar application of HA is beneficial in production, as it can increase the economic yield of naked oats. However, the increase in panicle biomass during the grain-filling stage inhibits the enhancement of β-glucan content in the grains.

## 5. Conclusions

Based on the result of the pool experiment, the interaction between HA and the irrigation amount does not significantly affect the β-glucan content in oat grains. The irrigation amount significantly influences the β-glucan content in the grains at 15 DPA; the β-glucan content under 60 mm irrigation was 4.36% W/W, which is 24% higher than that under 120 mm irrigation (*p* < 0.05). The β-glucan content in the grains decreased as the irrigation amount increased. The application of HA spray at the booting and anthesis stages resulted in increases at 5, 15, and 25 DPA in the β-glucan content (3.14% W/W, 4.13% W/W, and 4.51% W/W) of naked oats in the grains. However, the amplitude of the increase diminished over time, representing increases of 16% (*p* < 0.05), 9%, and 5% compared to the water spray. The effect of HA at the late growth stage is reflected in the increase in biomass in the panicle. The β-glucan content of grains at5 and 15 DPA, along with glucose in panicles at 20 DPA, directly influences β-glucan levels at the grain maturity stage. At 10 DPA, the distribution of sucrose in the leaves and panicles influences the soluble sugar content, which subsequently regulates the β-glucan content in the grains at 15 DPA. Specifically, the sucrose content in the leaves exerts a positive regulatory effect, while the sucrose content in the panicles exerts a negative regulatory effect.

## Data Availability

The datasets analyzed during the current study are available from the corresponding author on reasonable request.
